# Crystal structure of R-spondin 2 in complex with the ectodomains of its receptors LGR5 and ZNRF3^☆^

**DOI:** 10.1016/j.jsb.2015.05.008

**Published:** 2015-08

**Authors:** Matthias Zebisch, E. Yvonne Jones

**Affiliations:** Division of Structural Biology, Henry Wellcome Trust Centre for Human Genetics, University of Oxford, Oxford OX3 7BN, United Kingdom

**Keywords:** Wnt agonist, RING finger ubiquitin ligase, Complex crystal structure, Stem cells, Signalling

## Abstract

The four secreted R-spondin (Rspo1-4) proteins of vertebrates function as stem cell growth factors and potentiate canonical Wnt signalling. Rspo proteins act by cross-linking members of two cell surface receptor families, complexing the stem cell markers LGR4-6 with the Frizzled-specific E3 ubiquitin ligases ZNRF3/RNF43. The consequent internalisation of the ternary LGR–Rspo–E3 complex removes the E3 ligase activity, which otherwise targets the Wnt receptor Frizzled for degradation, and thus enhances Wnt signalling. Multiple combinations of LGR4-6, Rspo1-4 and ZNRF3/RNF43 are possible, implying the existence of generic interaction determinants, but also of specific differences in complex architecture and activity. We present here a high resolution crystal structure of an ectodomain variant of human LGR5 (hLGR5_ecto_) complexed with a signalling competent fragment of mouse Rspo2 (mRspo2_Fu1-Fu2_). The structure shows that the particularly potent Rspo2 ligand engages LGR5 in a fashion almost identical to that reported for hRSPO1. Comparison of our hLGR5_ecto_ structure with previously published structures highlights a surprising plasticity of the LGR ectodomains, characterised by a nearly 9° or larger rotation of the N-terminal half of the horseshoe-like fold relative to the C-terminal half. We also report a low resolution hLGR5–mRspo2_Fu1-Fu2_–mZNRF3_ecto_ ternary complex structure. This crystal structure confirms our previously suggested hypothesis, showing that Rspo proteins cross-link LGRs and ZNRF3 into a 2:2:2 complex, whereas a 1:1:1 complex is formed with RNF43.

## 1 Introduction

Signalling by the Wnt family of secreted glycolipoproteins is considered to be one of the most fundamental developmental signalling pathways and occurs in all animals that share a primary body axis (Holstein, 2012). Due to its crucial roles in embryonic development and tissue homeostasis it is subject to a multi-layered system of regulation (Clevers and Nusse, 2012; Niehrs, 2012; Malinauskas and Jones, 2014). A multitude of Wnt antagonists are secreted which function by blocking receptor access (Semenov et al., 2001; Mao et al., 2001; Semenov et al., 2005), sequestering Wnt ligands (Wang et al., 1997; Leyns et al., 1997; Malinauskas et al., 2011) or degrading Wnt (Zhang et al., 2012; Kakugawa et al., 2015; Zhang et al., 2015). In contrast, R-spondins (Rspo1-4) are the sole secreted potentiators of Wnt signalling (Kazanskaya et al., 2004) and have emerged as crucial regulators of stem cell maintenance *in vivo* and *in vitro* (Sato et al., 2009; de Lau et al., 2014). These stem cell growth factors are found in regenerating tissue such as nail mesoderm (Blaydon et al., 2006), hair follicle (Cadieu et al., 2009) and most prominently in the intestinal epithelium (Kim et al., 2005). Structurally they consist of two cysteine rich repeats (similar to those found in Furin and henceforth referred to as Fu1 and Fu2, respectively), which are necessary and sufficient for Wnt activation, as well as a C-terminal Thrombospondin-related domain (TSR) and a positively charged, flexible tail.

At the level of molecular mechanism Rspo proteins potentiate Wnt signalling by alleviating the feedback inhibition effected by the transmembrane E3 ligases ZNRF3 and RNF43 (de Lau et al., 2014). These two homologs whose expression is up-regulated by canonical Wnt signalling have been found to specifically target the cell surface Wnt receptors of the GPCR-like Frizzled family for degradation (Koo et al., 2012; Hao et al., 2012). Rspo1-4 bind to the extracellular PA domain (from *protease associated*) of these E3 ligases with different affinities which are correlated to their potency in cell-based assays (Kim et al., 2008; Zebisch et al., 2013). Although necessary, binding of Rspo to the E3 ectodomains is not sufficient to stop ubiquitination of Frizzled and enhance Wnt signalling. This further requires simultaneous binding of Rspo to the large leucine-rich repeat (LRR) containing ectodomain of LGR receptors 4, 5 or 6, which are well established GPCR-like regulators of stem cell function (Carmon et al., 2011; de Lau et al., 2011; Glinka et al., 2011; Ruffner et al., 2012; Xie et al., 2013). The ternary complex between Rspo and its two receptors LGR4-6 and ZNRF3/RNF43 is established via extracellular recognition and triggers by an unknown mechanism its rapid internalisation. This results in efficient removal of the E3 ligases from the cell surface and hence increases Wnt signalling through Frizzled.

A plethora of crystal structures of Rspo alone and in complex with LGR4 or -5, ZNRF3 or RNF43 were published in 2013 (Wang et al., 2013; Chen et al., 2013; Zebisch et al., 2013; Peng et al., 2013a; Peng et al., 2013b), defining key elements of the LGR–Rspo–E3 complex only two years after the identification of this elaborate mechanism for potentiating Wnt signalling. All LGR complex structures published to date (Xu et al., 2013; Wang et al., 2013; Chen et al., 2013; Peng et al., 2013a) contain Rspo1 as binding partner rather than one of the more potent Rspo2 or -3 ligands (Kim et al., 2008; Zebisch et al., 2013; Warner et al., 2015). One paper described the crystal structure of a ternary LGR5–RSPO1–RNF43 complex that displayed a clear 1:1:1 stoichiometry (Chen et al., 2013). However, a dimeric arrangement of ZNRF3_ecto_ was found for multiple independent structure determinations of complexes with hRSPO1 and Rspo2 as well as in most of the unliganded crystal structures (Zebisch et al., 2013; Peng et al., 2013b). We found that dimerisation was stabilized by Rspo ligand binding, but also enhanced the ligand binding affinities (Zebisch et al., 2013). These observations prompted us to suggest that ternary complexes involving ZNRF3 would have a 2:2:2 stoichiometry.

In order to further advance our understanding of the functional properties of LGR–Rspo–E3 complexes we sought to detail the interaction interface of the more potent Rspo2 ligand with hLGR5_ecto_ and to test our hypothesis concerning the stoichiometry of the ternary complex. We describe here a high resolution structure of hLGR5_ecto_ in complex with mRspo2_Fu1-Fu2_ and analyse the ligand binding interface and receptor structural plasticity. Furthermore we describe the structure of a ternary hLGR5–mRspo2_Fu1-Fu2_–mZNRF3_ecto_ complex solved at 5 Å resolution.

## 2 Methods and materials

### 2.1 Large scale mammalian expression and protein purification

Mouse Rspo2_Fu1-Fu2_ and ZNRF3_ecto_ proteins were produced as described (Zebisch et al., 2013). A cDNA clone for human LGR5 was obtained from the I.M.A.G.E. library. The ectodomain of LGR4, -5, and -6 contains an unstructured loop just before the last β strand of the horseshoe fold (in hLGR5 between C485 and V539). This region forms in part a helical structure in the single PDB entry 4BSR. The relevance of this observation is however unclear due to potentially stabilizing crystal packing interactions and the low resolution of the structure. Before crystal structures of LGR ectodomains were reported, we tested, but struggled to achieve high level secretory expression for many LGR ectodomain constructs. We only managed to obtain expression in experiments with coexpression of a well expressing Rspo ligand. On inspection of the first crystal structures, we replaced the region A488-H537 (Chen et al., 2013; Peng et al., 2013a) by overlapping two step PCR with a short NGNNGD linker and cloned the ectodomain variant (R32-S543) into pHLsec H10 (Aricescu et al., 2006). The mature hLGR5_ecto_ protein after removal of the signal peptide therefore had the sequence ETG-RGCPTH...FGVCEN-NGNNGD-SVQCSPS-GTHHHHHHHHHH (natural residues underscored). Removal of the unstructured loop boosted the yield of expressed protein by least ten fold compared to that obtained in earlier trials. A complex of mRspo2_Fu1-Fu2_ (I39-G144, mature sequence ETG-ICKGCL...MECVEG-THHHHHHHHHH) with hLGR5_ecto_ was obtained from cotransfection of HEK293T cells cultured in the presence of 1 mg/l kifunensine (Cayman Chemicals). Approximately 5 mg of complex could be obtained from 1 l of mammalian cell culture. The dimeric protein complex was purified as previously described for the mRspo2_Fu1-Fu2_–mZNRF3_ecto_ complex (Zebisch et al., 2013) but using 10 mM Tris/HCl pH 9.0, 250 mM NaCl as running buffer during gel filtration.

### 2.2 Crystallisation and data collection

The hLGR5_ecto_–mRspo2_Fu1-Fu2_ complex was concentrated to 5.9 mg/ml and subjected to sitting drop vapour diffusion crystallisation trials. A single crystal appeared after one month in 25% (w/v) Polyethylene Glycol 3350, 200 mM Lithium Sulphate, 100 mM bis-Tris pH 5.5. For cryoprotection the crystal was transferred to reservoir solution to which 1/10 V PEG200 had been added before dipping into liquid nitrogen.

To obtain the hLGR5_ecto_–mRspo2_Fu1-Fu2_–mZNRF3_ecto_ complex we mixed approximately stoichiometric amounts of the hLGR5_ecto_–mRspo2_Fu1-Fu2_ complex and mZNRF3_ecto_ in 10 mM Tris/HCl pH 9.0, 1000 mM NaCl and adjusted the protein concentration to 1.3 mg/ml. The presumed complex was subjected to sitting drop vapour diffusion crystallisation trials mixing 300 nl of protein with 100 nl reservoir. Crystals appeared after 10 days in 100 mM Ammonium Acetate, 600 mM NaCl, 50 mM MES pH 6.0, 5 mM MgSO_4_ and grew slowly over the course of three months. For cryoprotection of these crystals the liquid around the crystals *in situ* was exchanged six times to new liquid with linearly increasing salt and glycerol content. The final cryoprotection solution was 100 mM Ammonium Acetate, 3 M NaCl, 10% glycerol, 50 mM MES pH 6.0, 5 mM MgSO_4_.

### 2.3 Structure determination

Diffraction data were collected at DIAMOND synchrotron light source at the i04 beamline. The structure of the high resolution hLGR5_ecto_–mRspo2_Fu1-Fu2_ complex was solved by Molecular Replacement (MR) using the previously described hLGR5_ecto_–hRSPO1_Fu1-Fu2_ complex as a starting model (Peng et al., 2013a).

For the ternary complex we obtained only a low resolution, highly anisotropic dataset extending in the best direction to 4.8 Å (*I*/*σI *= 2). The asymmetric unit contains a single hLGR5_ecto_–mRspo2_Fu1-Fu2_–mZNRF3_ecto_ complex. The 2:2:2 complex as described in the Results section is generated by crystallographic symmetry. To solve this structure we proceeded as follows. Structure determination by MR was facilitated by anisotropic scaling via the anisotropic diffraction server at the UCLA MBI (http://services.mbi.ucla.edu/anisoscale/) which reported recommended resolution limits along *a*^∗^, *b*^∗^ and *c*^∗^ to be 5.7, 5.7, and 4.8 Å. We first tested all available LGR5_ecto_ structures for the highest score in MR using the PHASER program. The best model appeared to be chain A of PDB entry 4BSU (Peng et al., 2013a) for which we obtained a single solution with a Translation Function Z score (TFZ) of 12.6 and a log likelihood gain (LLG) of 393 indicating a clear solution. With hLGR5_ecto_ in place we then compared MR scores for all available mZNRF3_ecto_ structures (Zebisch et al., 2013; Peng et al., 2013b) and found chain C of PDB entry 4C99 (Zebisch et al., 2013) to give the highest score (TFZ = 9.1, combined LLG = 573). After rigid body refinement in REFMAC strong density was apparent between the concave side of hLGR5_ecto_ and mZNRF3. mRspo2_Fu1-Fu2_ could not be placed by MR likely because the exact conformation of the small cysteine knot protein was not sampled by the available structures. We hence placed Fu1 and Fu2 individually by superposing Fu1-ZNRF3 and Fu2-LGR5 complexes onto the MR solutions. The generated hLGR5_ecto_–mRspo2_Fu1_–mRspo2_Fu2_–mZNRF3_ecto_ complex was subjected to rigid body refinement. As we observed a deviation of the model from the electron density map between β-hairpins 2 and 3 of Fu1, we defined two rigid bodies for Rspo2: I39-L75 and H76-C141. The geometry of L75-S77 was afterwards idealised in COOT. We then performed restrained refinement of the coordinates against the original (uncorrected) diffraction data using strong geometric restraints generated by PROSMART from the available high resolution reference structures. The single restrained refinement step (20 cycles) was followed by 10 cycles of structure idealisation. The final model had an *R*_work_ and *R*_free_ of 27.0% and 31.3%, respectively.

### 2.4 Structural analysis

Superpositions of LGR ectodomains were performed with the ALIGN program as implemented in PYMOL. Domain and hinge identification was performed using DYNDOM with standard settings. Figures were produced in PYMOL and assembled in PHOTOLINE32.

## 3 Results

### 3.1 The LGR5–Rspo2 binding interface

**Fig. 1:**
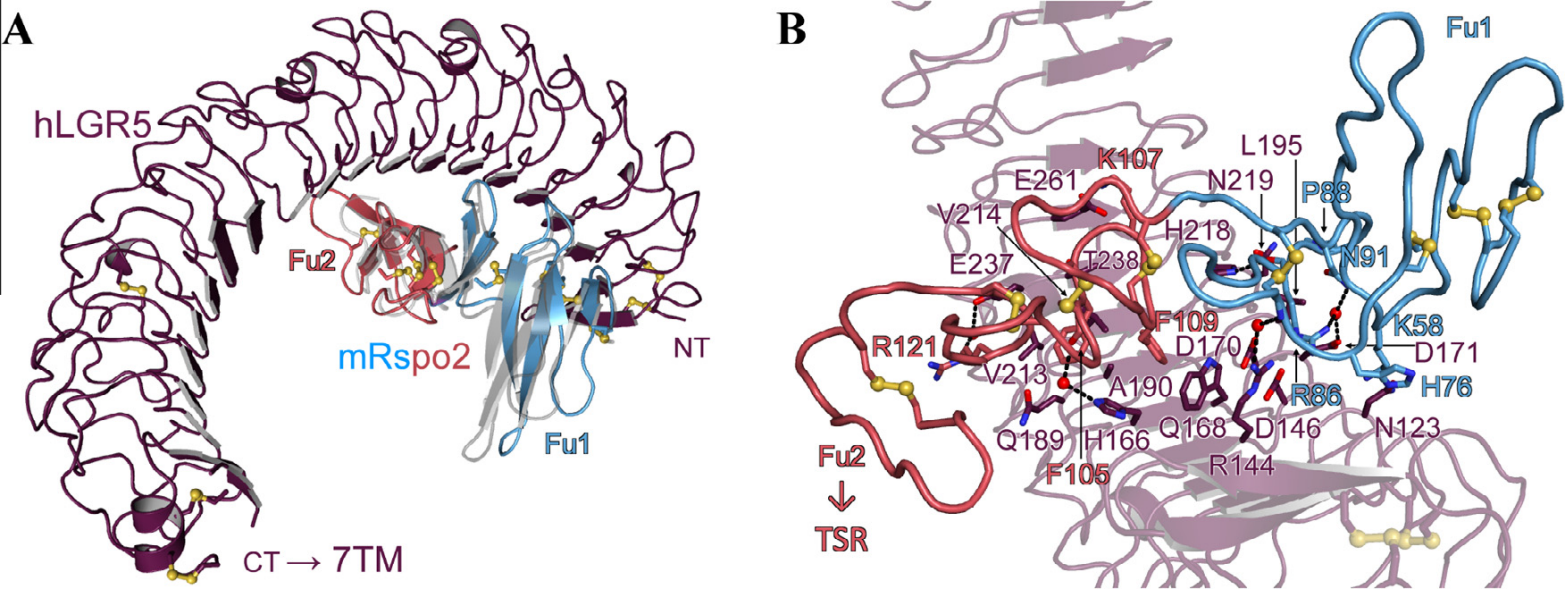
The hLGR5–mRspo2 binding interface in two views. (A) Overview of the binding interface. Only the N-terminal half of the LGR5 horseshoe fold is involved in ligand binding. The relative orientation of hRSPO1 from PDB entry 4KNG was generated by superposing the N-terminal region of LGR5 (residues 96-308) and is displayed in gray. (B) Close-up view on the binding site. Cysteine bridges are shown as yellow ball-and-sticks and dotted lines represent hydrogen bonds.

To provide insight into recognition of Rspo2 by LGR receptors we crystallised a variant of the hLGR5 ectodomain (from which an unstructured loop had been removed, see Methods) in complex with the Furin tandem repeat of mRspo2 (Table 1, Fig. 1). The crystal structure was solved at 2.2 Å and refined to *R*_work_ and *R*_free_ values of 20.9% and 26.0%. The asymmetric unit contains two copies of the hLGR5_ecto_–mRspo2_Fu1-Fu2_ complex, which are essentially identical (r.m.s.d. of 0.5 Å for 559 aligned Cα). A structural superposition with the corresponding highest resolution complex of hLGR5_ecto_–hRSPO1_Fu1-Fu2_ (PDB entry 4KNG (Chen et al., 2013)) reveals that almost identical residues are involved in the molecular recognition. At the core of the interface is a hydrophobic interaction site with the hallmark ‘phenylalanine clamp’ (Xu et al., 2013; Wang et al., 2013; Chen et al., 2013; Peng et al., 2013a), in this example F105 and F109 of Rspo2_Fu2_ pinching A190 of hLGR5. These conserved hydrophobic core interactions are surrounded by more polar interactions which differ in a subtle way from the hLGR5–hRSPO1 interface (not all shown in figures): The side chain of N123 is found to form a hydrogen bond with H76, an interaction that was not previously seen. The salt bridge seen between R144 of LGR5 and D85 of hRSPO1 is replaced with a water-mediated hydrogen bond to the backbone of C78 of mRspo2. In the hLGR5–hRSPO1 complex a further two hydrogen bonds are formed between Q189 and T238 to G123 and N109 respectively. These two hydrogen bonds are not found in the complex described here. The overall similarity in the complex structures is in agreement with the promiscuity of Rspo–LGR binding and the observation that all Rspo ligands bind LGR receptors with similar nanomolar affinity and potency being largely determined by interaction with the E3 ligases (Carmon et al., 2011; Zebisch et al., 2013).

### 3.2 A recurring LGR5 ectodomain dimer

**Fig. 2:**
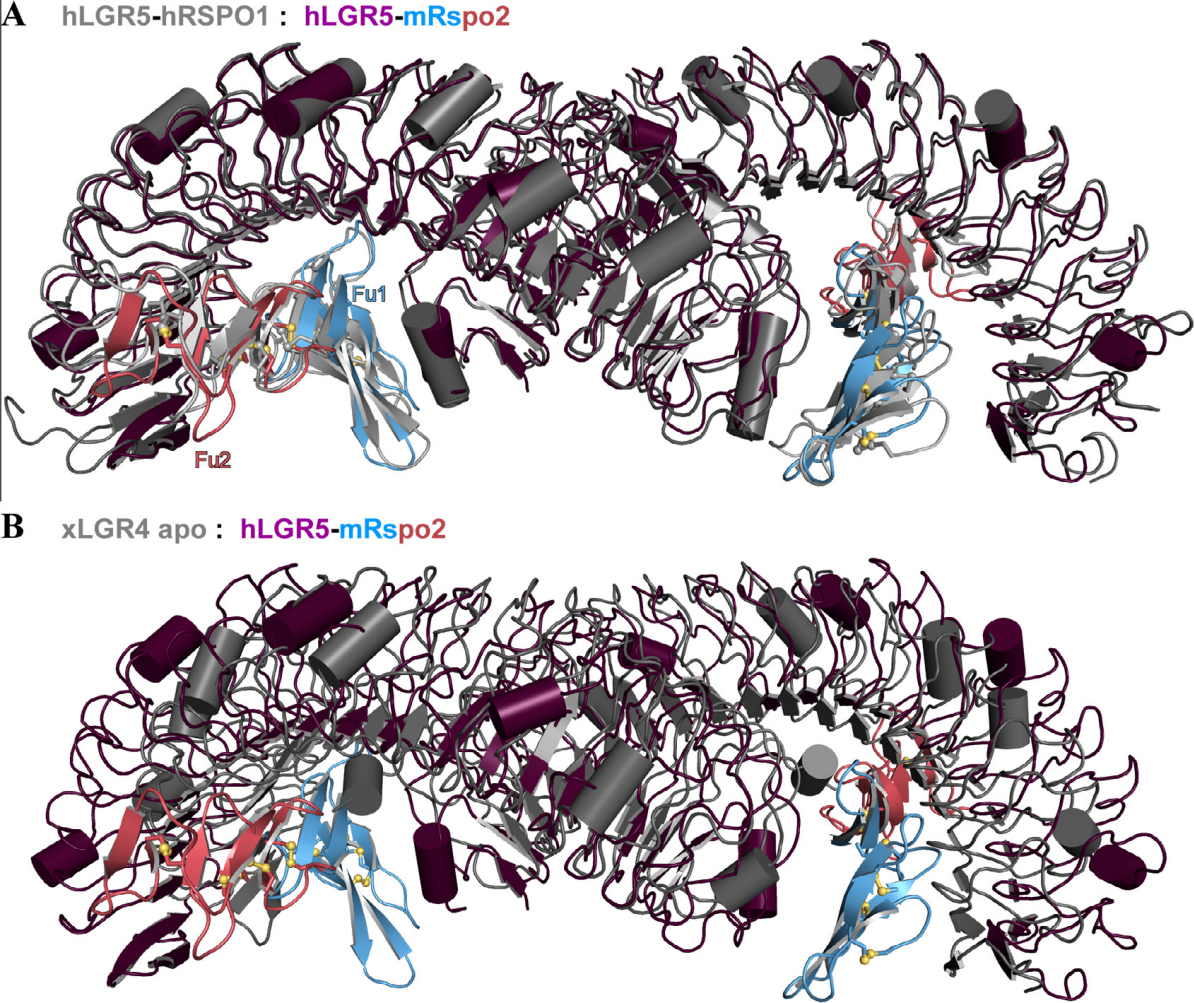
A recurring dimeric arrangement of LGR receptor ectodomains. Superposition of hLGR5_ecto_–mRspo2_Fu1-Fu2_ (coloured) with the hLGR5_ecto_–hRSPO1_Fu1-Fu2_ complex (gray, PDB entry 4BSR) in A and with the xLGR4_ecto_ apo structure (gray, PDB entry 4LI1) in B.

For all four of their published crystal structures of hLGR5_ecto_–hRSPO1_Fu1-Fu2_ complexes Peng et al. observed an identical packing interaction between neighbouring hLGR5 ectodomains in otherwise different crystal lattices (Peng et al., 2013a). These observations initially prompted the suggestion that there may be a recurring 2:2 stoichiometry for the LGR–Rspo interaction, however, subsequent structures have revealed a confusingly diverse set of LGR–LGR packing interactions rather than one consistent quaternary arrangement (de Lau et al., 2014). We observe the exact same dimeric arrangement with a twofold rotational symmetry in our structure as that seen by Peng et al. (Fig. 2A). The fact that all five crystal structures are crystallographically unrelated and resulted from diverse crystallisation conditions points to a high propensity of the hLGR5 ectodomain to dimerize in this particular fashion. This tendency to dimerize might even be higher in the native membrane as the proteins would have fewer degrees of motional freedom. The LGR5–LGR5 interface buries some 900 Å of solvent accessible area. Residues involved in the dimer interface include Y289, D290, Y361, H383, W407, H454 which interact in a rather loose fashion, as judged from visual inspection of shape complementarity (not shown), and are only partially conserved across LGR4-6. Most notably however, we observe a very similar dimer arrangement in the crystal structure of *Xenopus* (x) LGR4_ecto_ (Fig. 2B) pointing towards a conserved dimerisation mode. The observed dimeric arrangement is compatible with simultaneous anchoring in *cis* to the same cell membrane (i.e. both C-termini point in the same direction). However, as has been noted earlier, this arrangement is not compatible with simultaneous binding of RNF43 or ZNRF3 to Rspo (Zebisch et al., 2013; Peng et al., 2013b). It remains to be established whether disruption of ectodomain dimerisation may for the full integral membrane LGR receptor be in some way involved in signal transmission by R-spondins.

### 3.3 Structural plasticity of LGR ectodomains

**Fig. 3:**
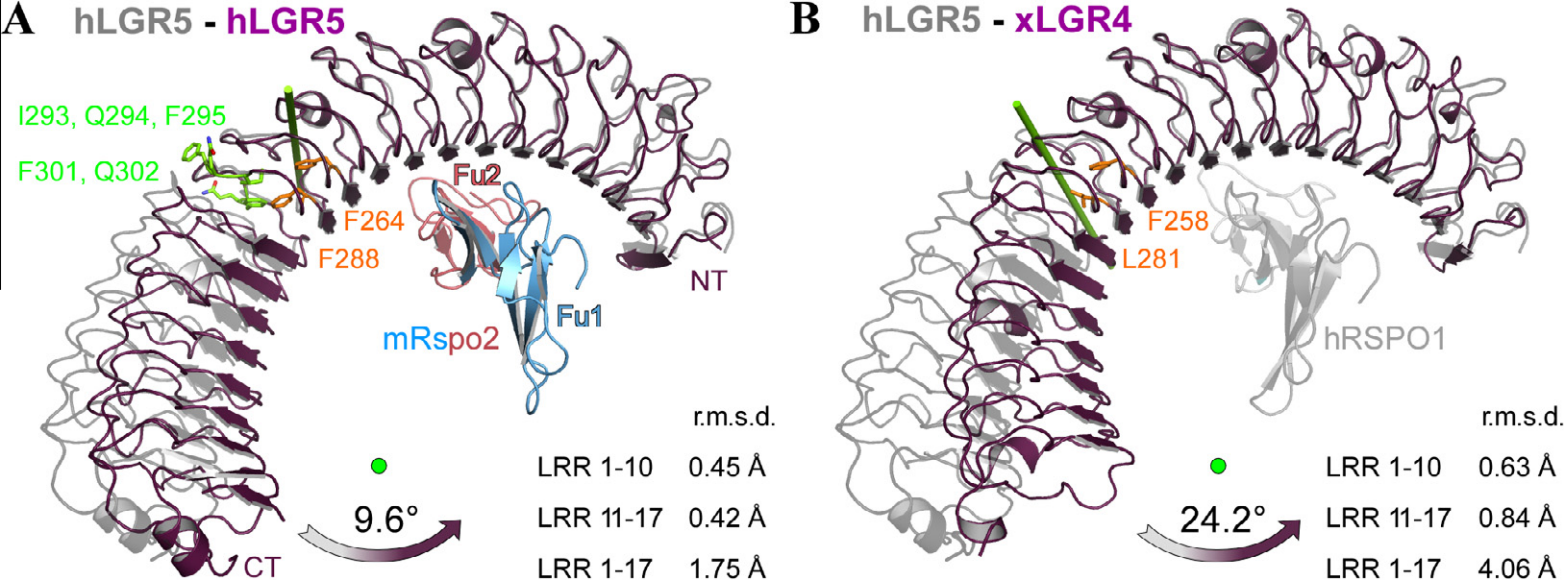
A hinge motion in LGR5 receptor ectodomains. (A) Superposition of the hLGR5_ecto_–mRspo2_Fu1-Fu2_ complex (this work, coloured) with hLGR5_ecto_–hRSPO1_Fu1-Fu2_ (pdb 4BSU, gray, hRSPO1 not shown) based on the 10 N-terminal LRR. The C- and N-terminal regions can rotate as separate rigid bodies almost 10° around the axis shown as green rod. Calculated hinge residues are shown in green. Two phenylalanine side chains occupying canonical leucine positions are shown in orange. (B) A similar comparison of hLGR5_ecto_–hRSPO1_Fu1-Fu2_ (pdb 4BSU, gray) to the apo form of xLGR4_ecto_ (pdb: 4LI1) indicates that the two segments of LGR ectodomains could undergo domain movements of up to 24°.

During our structural comparisons of the herein described hLGR5_ecto_–mRspo2_Fu1-Fu2_ complex with previously published LGR_ecto_ structures we noticed surprisingly high r.m.s.d. values for aligned Cα traces. As an example, superposition of the two LGR chains of the complex described here to chain E of PDB entry 4BSU (hLGR5_ecto_–hRSPO1_Fu1-Fu2_ complex, that is 100% sequence identity) resulted in an r.m.s.d. values of 1.8 and 1.9 Å, respectively. The ectodomain structure of xLGR4 gave even higher r.m.s.d. values of up to 4.1 Å (56% sequence identity). Such high structural deviations indicate structural rearrangements, i.e. domain movements. We noticed that when the N-terminal or C-terminal regions were superimposed separately the r.m.s.d. values achieved were considerably lower (Fig. 3). For an accurate and quantitative analysis we employed the program DYNDOM as implemented in CCP4 (Hayward and Berendsen, 1998), which allows the identification of domain borders, hinge regions and associated domain rotation axes based on comparison of different crystal structures.

In the case of LGR5 using the structure described here and chain E of PDB entry 4BSU the program identified two domains (Fig. 3A). Domain 1 (P35-F301) encompasses the first 10 LRR and domain 2 (Q302-C541) consists of LRR 11-17. Residues I293, Q294, F295, F301 and Q302 function as hinge regions mediating a domain rotation of 9.6° around an axis approximately perpendicular to the horseshoe fold. It is noticeable that the rotation axis and hinge region is very close to residues F264 and F288 which have been noticed earlier to disrupt the LRR fold (Peng et al., 2013a). Hence the presence of these residues, at what are canonical leucine positions in the LRR sequence motif, likely contributes to the observed dynamic properties. The structure described here represents the most tightly curved or concave conformation observed to date for the LGR5 ectodomain. However, the ectodomain of LGR4 (Xu et al., 2013) corresponds to an even more closed horseshoe. A comparison of LGR5 and LGR4 ectodomains suggests that a LGR receptor may be capable of rotational flexion of up to 24° within its extracellular region (Fig. 3B). This flexion does not appear to provide an induced fit-type mechanism for binding to Rspo ligands. However, it may contribute an element of adaptability needed for the formation multi-subunit complexes with the various combinations of Rspo ligands and ZNRF3/RNF43 ligases or other as yet uncharacterised binding partners.

### 3.4 Architecture of the LGR5–Rspo–ZNRF3 complex

**Fig. 4:**
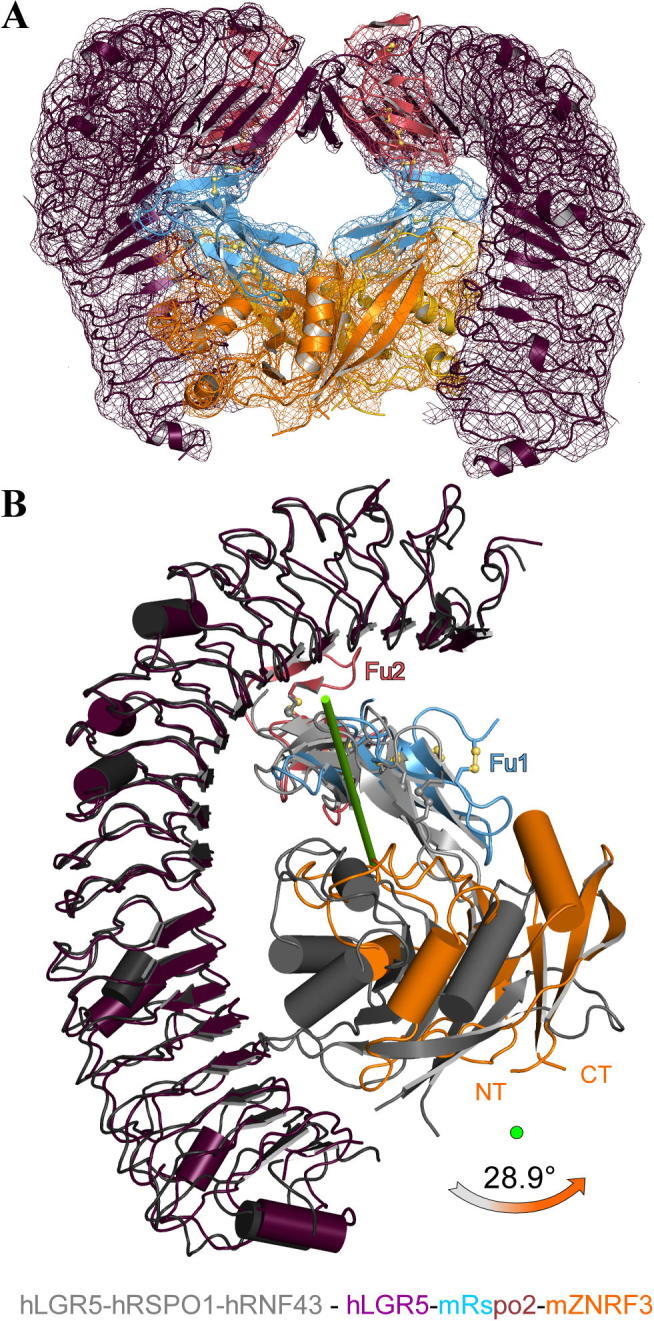
Architecture of the ternary LGR–Rspo–ZNRF3 signalling complex. (A) The ternary hLGR5_ecto_–mRspo2_Fu1-Fu2_–mZNRF3_ecto_ complex adopts a 2:2:2 stoichiometry in the crystal lattice. LGR5 and ZNRF3 do not contact each other directly. The 2F_o_–F_c_ density, depicted as chicken wire, is contoured at the 1*σ* level (mRspo2_Fu1-Fu2_ blue-pink; hLGR5_ecto_ magenta; mZNRF3_ecto_ orange/yellow). (B) Comparison of the two ternary LGR–Rspo–E3 complexes (1:1:1 complex for each) superimposed based on hLGR5. Compared to the RNF43 complex (in gray) ZNRF3 (orange) together with the Fu1 unit of Rspo2 rotates almost 30° around the axis shown in green. This is required to avoid steric clashes in the membrane-proximal part of the two receptors that would result from 2:2:2 complex formation (not shown).

A multitude of crystal structures of Rspos with either LGR or ZNRF3/RNF43 ectodomains have been published (Xu et al., 2013; Wang et al., 2013; Chen et al., 2013; Zebisch et al., 2013; Peng et al., 2013a; Peng et al., 2013b). Only a single structure describes the architecture of Rspo in a ternary complex with ectodomains of both types of receptors, that is the hLGR5_ecto_–hRSPO1_Fu1-Fu2_–hRNF43_ecto_ complex (Chen et al., 2013). To obtain insight into ternary complex formation with ZNRF3 we solved a low resolution hLGR5_ecto_–mRspo2_Fu1-Fu2_–mZNRF3_ecto_ complex (see methods, Table 1 and Fig. 4). This complex is very similar to the equivalent RNF43-containing complex in the overall positioning of the three different types of subunits. The Rspo_Fu1-Fu2_ fragment is sandwiched between the two receptor ectodomains which do not contact each other (Fig. 4A). Thus, a Rspo ligand serves to heterodimerize an LGR receptor with an E3 receptor in both flavours of ternary complex. However, as previously proposed by us (Zebisch et al., 2013) the ternary complex involving ZNRF3 adopts a clear 2:2:2 stoichiometry in the crystal lattice due to dimerisation of the ZNRF3 ectodomain. Compared to the relative position of RNF43 in the ternary complex, each ZNRF3 subunit is rotated 30° away from its partner LGR5 (Fig. 4B). We note that dimerisation of ZNRF3_ecto_ and consequent 2:2:2 stoichiometry of the ternary complex would not be compatible with the exact arrangement of ligand and receptors as seen in the RNF43 ternary complex due to clashes of the membrane-proximal regions of the two LGR receptors (not shown). This rigid body shift in the position of the ZNRF3/RNF43 relative to LGR also requires a re-orientation of the Rspo2_Fu1_ domain to maintain the β-hairpin 1 and 2 mediated interface with the E3 (Chen et al., 2013; Zebisch et al., 2013; Peng et al., 2013b). The flexibility of the Rspo Fu1-Fu2 linker has been previously noted (Zebisch et al., 2013; Peng et al., 2013a). Here we observe a slight twist before the third β-hairpins of Fu1 consistent with it forming part of the mostly involved in LGR binding (Cheng et al., 2011; Xu et al., 2013; Wang et al., 2013; Peng et al., 2013a). It appears that the difference in relative orientation of the LGR and E3 receptors for the RNF3 versus ZNRF43 ternary complex renders this flexibility a necessity for Rspo to function as a cross-linker in both contexts.

## 4 Discussion and conclusions

R-spondins are important stem cell growth factors with reported roles in tissue regeneration, but also cancer progression (Kim et al., 2005; Zhao et al., 2009; Seshagiri et al., 2012; Papapietro et al., 2013). An understanding of the basic principles behind R-spondin signalling is fundamental to the design of novel therapeutic interventions for cases where the Wnt pathway is deregulated. We provide here detailed insights into the recognition of Rspo2 by LGR5. These insights can inform the development of potency-enhanced artificial Rspo molecules (Moad and Pioszak, 2013; Warner et al., 2015) for example, to enhance tissue regeneration. Conversely, our mapping of the interaction determinants can also facilitate the design of Rspo antagonistic binding molecules, with potential applications in fighting Wnt driven cancers.

Our crystallographic analysis has added to the previously reported observations that LGR receptor ectodomains have a propensity to dimerize in a specific symmetrical fashion (Peng et al., 2013a; de Lau et al., 2014). Despite the multiple examples of this behaviour occurring in otherwise unrelated crystal packing arrangements, the functional relevance of LGR receptor homo-dimerisation remains speculative for now. One possibility is that disruption of the LGR dimer during formation of LGR–Rspo–E3 complexes may be involved in R-spondin signal transmission.

It has previously been noted that the horseshoe fold of LGR ectodomains is partially split into two segments (Wang et al., 2013; Peng et al., 2013a). We have shown here for the first time that the N-terminal and C-terminal regions behave as separate rigid bodies, i.e. domains that can undergo relative domain motions of up to 10°, and possibly as much as 24°. Only the N-terminal 10 LRRs are involved in ligand binding. What is the function of the C-terminal region of the horseshoe fold? This region has a slightly lower sequence conservation across homologs as compared to the ligand binding domain. It may well be that the sole function of this region is to provide a sturdy linker between the ligand binding region and the transmembrane domain so as to place tight limits on the relative positions of the transmembrane and cytosolic regions for R-spondin cross-bridged E3 ligases and LGR receptors. An additional possibility is that this region harbours determinants for association with the Wnt receptors Frizzled and LRP5/6 (de Lau et al., 2011; Carmon et al., 2012).

Our crystallographic LGR5–Rspo–ZNRF3 complex, albeit at low resolution, confirms the 2:2:2 stoichiometry that we had hypothesised would be favoured for ternary complexes involving ZNRF3 (Zebisch et al., 2013), as opposed to the 1:1:1 stoichiometry seen for the corresponding RNF43 complex (Chen et al., 2013). This contrast in ternary complex composition was unsuspected given the apparent similarity in sequence and function of ZNRF3 and RNF43. However, the difference in dimerisation potential between the two E3 ligase ectodomains is apparent on detailed structural analysis. RNF43 lacks the structural determinants that favour dimerisation in ZNRF3, and ZNRF3 dimerisation is strongly enhanced by Rspo ligand binding (Zebisch et al., 2013). This clear difference in the behaviour of two, modestly conserved, E3 ligases calls for further studies to uncover functional differences and their physiological implications.

## Conflict of interest

The authors declare no competing financial interests.

## Figures and Tables

**Table 1:** Data collection and refinement statistics.

PDB code	4UFR	4UFS
Complex	hLGR5_ecto_–mRspo2_Fu1-Fu2_	hLGR5_ecto_–mRspo2_Fu1-Fu2_–mZNRF3_ecto_
Stoichiometry	1:1	2:2:2 (1:1:1 per AU)
*Data collection*		
X-ray source	i04	i04
Wavelength (Å)	1.0088	0.9795
Space group	*C*2	*I*422
*a*, *b*, *c* (Å)	205.2, 59.8, 112.2	188.6, 188.6, 165.3
*α*, *β*, *γ* (°)	90, 99.5, 90	90, 90, 90
Wilson B-factor (Å^2^)	37	212
Resolution range (Å)	69.19–2.20 (2.26–2.20)	39.47–4.80 (5.37–4.80)
Unique reflections	62167 (2968)	7509 (2105)
Average multiplicity	7.6 (2.6)	5.9 (6.1)
Completeness (%)	90.6 (59.6)	99.4 (99.8)
〈*I*/*σI*〉	10.8 (1.2)	8.5 (1.5)
*R*_merge_ (all) (%)	12.5 (68.5)	16.1 (153.2)
*R*_pim_ (all) (%)	4.6 (45.0)	7.1 (67.8)

*Refinement*		
*R*_work_/*R*_free_ (%)	20.9/26.2	27.0/31.3
No. of non H atoms		
Protein	8756	5582
Water	150	–
Ligands	6	–
Average B-factor (Å^2^)		
Protein	62	258
Water	47	–
Ligands	63	–
r.m.s.d. from ideality		
Bond lengths (Å)	0.0137	0.004
Bond angles (°)	1.7460	0.848
Ramachandran plot		
Favoured (%)	90.7	90.7
Allowed (%)	99.9	99.4
Outliers (number)	1	4

Values in parentheses are for the highest-resolution shell.
